# Comprehensive Phytohormone Analysis Reveals the Roles of Auxin, Cytokinin, and Gibberellin in Enhancing Seed Germination and Growth of *Chimonobambusa utilis*

**DOI:** 10.3390/plants14243780

**Published:** 2025-12-11

**Authors:** Wanqi Zhao, Simei Ai, Haixiang Yuan, Mingzhen Lv, Shuyan Lin

**Affiliations:** State Key Laboratory for Development and Utilization of Forest Food Resources, Co-Innovation Center for Sustainable Forestry in Southern China, Bamboo Research Institute, College of Forestry and Grassland, Nanjing Forestry University, Nanjing 210037, China; wanqzhao@163.com (W.Z.);

**Keywords:** *Chimonobambusa utilis*, seed germination, plant growth regulators, seedling growth, propagation optimization

## Abstract

Bamboo seeds (often called bamboo rice) are nutritionally rich, offering protein, fiber, and essential minerals like potassium and manganese. *Chimonobambusa utilis* seeds, especially, represent an underexplored nutritional resource with exceptional edible and agricultural potential. Here, we report that *Ch. utilis* seeds contain remarkably high levels of unsaturated fatty acids (67.39% of total lipids), with linoleic and linolenic acids comprising 36.5% and 26.7%, respectively, exceeding major vegetable oils by 1.5 to 3.3-fold. Comprehensive plant growth regulator (PGR) screening revealed distinct regulatory patterns: gibberellic acid (GA_3_, 8.66 µM) exhibits biphasic dose–response kinetics, cytokinins (6-BA, 222.0 µM) show nonlinear responses transitioning from low-concentration inhibition to high-concentration promotion with preferential lateral root induction, while auxins (NAA, 134.2 µM) demonstrate unimodal responses with concentration-dependent efficacy, achieving the strongest root-promoting effect (27% increase, *p* < 0.05). Mechanistically, optimal phytohormone treatments sustained elevated soluble sugar levels and differentially modulated key enzymes. Notably, 6-BA potently suppressed sucrose synthase activity while NAA maximally stimulated starch biosynthetic enzyme activities (AGPase and GBSS), identifying sucrose metabolism as a pivotal regulatory node. Comparative evaluation of germination capacity and seedling vigor revealed that individual treatments with 8.66 µM GA_3_, 222.0 µM 6-BA, or 134.2 µM NAA achieved the best performance among tested concentrations, reducing germination time by 5 days and increasing germination percentage by 4.2 to 6.3% relative to control. These findings establish *Ch. utilis* as a premium oil crop candidate and provide mechanistic insights into phytohormone-mediated germination control with broad implications for bamboo seed biology and propagation optimization.

## 1. Introduction

Seeds represent a critical evolutionary advancement that have enabled seed plants to colonize diverse terrestrial environments through enhanced dispersal capacity and dormancy mechanisms [1,2]. As the fundamental unit of sexual reproduction in angiosperms and gymnosperms, seeds serve as repositories of genetic diversity and nutritional reserves that determine germination success and seedling establishment [3,4]. The transition from seed dormancy to active germination involves complex physiological and biochemical transformations, including the mobilization of storage compounds, hormonal regulation, and metabolic remodeling [5,6]. Understanding these processes is essential for developing effective strategies for seed-based propagation, particularly in species with recalcitrant seeds or prolonged flowering cycles.

Bamboos (*Bambusoideae*) constitute a unique group within Poaceae, characterized by extended vegetative phases punctuated by rare, often synchronous flowering events followed by mass mortality [7,8]. This monocarpic life history strategy poses significant challenges for bamboo conservation and utilization, as seed availability is highly unpredictable and often limited to narrow temporal windows [9,10,11]. Furthermore, many bamboo seeds exhibit recalcitrant storage behavior, losing viability rapidly under conventional storage conditions due to high moisture content and active metabolism [12,13,14,15]. These characteristics severely constrain research on bamboo sexual reproduction, genetic improvement, and large-scale regeneration programs. Among bamboo species, seeds can be classified into three morphological types: standard caryopses (e.g., *Phyllostachys edulis*) [16], nut-like caryopses (e.g., *Dendrocalamus latiflorus*) [17], and berry-like caryopses (e.g., *Chimonobambusa species*) [18]. The latter type, characterized by fleshy pericarp and high-water content, presents particular challenges for storage and germination management.

PGRs play pivotal roles in modulating seed germination through multiple mechanisms, including breaking dormancy, enhancing reserve mobilization, and promoting embryonic axis growth [19]. Gibberellins (GAs) are well-established germination promoters that antagonize abscisic acid signaling and activate hydrolytic enzymes responsible for mobilizing starch, proteins, and lipids from storage tissues [20,21]. Cytokinins, such as 6-benzylaminopurine (6-BA), enhance cell division and differentiation during germination and early seedling development, while also modulating stress responses [22,23]. Auxins, including α-naphthaleneacetic acid (NAA), regulate cell elongation and root initiation, although the germination-promoting effects of these PGRs are highly concentration-dependent [24,25]. Extensive research over the past several decades has established that optimal PGR treatments can significantly improve germination parameters in various species, including crops [26,27], ornamentals [28,29], and forest trees [30,31].

*Chimonobambusa utilis*, is an economically and ecologically important bamboo species endemic to the mountainous regions of southwestern China, particularly along the Dalou Mountain Range [32]. This species occupies high-altitude habitats (1000 to 2100 m elevation) and has expanded dramatically in recent decades, with cultivated areas exceeding 66,700 hectares in Tongzi County alone by 2020 [33]. *Ch. utilis* possesses multifaceted value encompassing nutrition, ecology, and economics. Its shoots are prized for their high protein content (12%), rich amino acid profile, and bioactive compounds including flavonoids and polysaccharides with demonstrated antioxidant, hepatoprotective, and immunomodulatory activities [34,35,36]. Despite its economic importance, research on *Ch. utilis* has predominantly focused on shoot quality [34,35], sheath utilization [37], and basic seed composition [38], with limited systematic investigation of seed germination physiology and seedling development. In recent years, southwestern China, including Guizhou, Chongqing, Yunnan, and Sichuan provinces, has vigorously developed the *Ch. utilis* industry, establishing millions of hectares of new plantations. Although the conventional practice of transplanting mother bamboo culms enables rapid forest establishment, the resulting stands exhibit unclear age structures and face potential risks of synchronous flowering, a phenomenon that can lead to widespread die-off in clonal bamboo populations. To mitigate these risks and enhance plantation sustainability, there is an urgent need to supplement existing stands with seedling-based regeneration by interplanting a proportion of seed-origin plants. Recent sporadic flowering events in natural *Ch. utilis* stands along the Dalou Mountains have provided opportunities to collect seeds for germination studies, yet optimal germination protocols and their underlying physiological mechanisms remain poorly defined.

The present study aimed to comprehensively characterize the nutritional composition of *Ch. utilis* seeds and elucidate the physiological mechanisms by which three major PGRs (GA_3_, 6-BA, and NAA) regulate seed germination and seedling growth. Specific objectives were to: (i) assess the nutritional quality of *Ch. utilis* seeds relative to other bamboo species and compare fatty acid profiles with common vegetable oils; (ii) identify effective concentrations of GA_3_, 6-BA, and NAA for promoting seed germination; (iii) investigate PGR-mediated changes in storage carbohydrate dynamics and related enzymatic activities during germination; (iv) evaluate PGR effects on morphological and physiological traits of emerging seedlings; and (v) conduct multifactorial comprehensive evaluation to identify the most effective PGR treatment regimes. By integrating germination kinetics, biochemical analyses, and seedling performance assessment, this research provides both fundamental insights into bamboo seed biology and practical protocols for *Ch. utilis* seedling production, contributing to the sustainable development of this valuable bamboo resource.

## 2. Materials and Methods

### 2.1. Plant Material Collection and Preparation

Seeds of *Ch. utilis* were collected in late April from Tongzi County, Zunyi City, Guizhou Province, China (coordinates: N 28°28′18″, E 107°2′43″; elevation: 1591 m). During the collection period, the site experienced predominantly cloudy weather conditions with average daily low temperatures of 12 °C and average daily high temperatures of 20 °C, with relative humidity of approximately 70–80%. Subsequent germination experiments were conducted from May to June under controlled laboratory conditions as described below. The collection site is characterized by a subtropical humid monsoon climate.

### 2.2. Morphological and Nutritional Characterization

Seed morphological parameters were determined using digital calipers on randomly selected seeds with and without glume. Seed length and width were measured, and the mass of 50 seeds was determined using an analytical balance (three replicates per treatment). Thousand seed weight was calculated by proportion and conversion.

To enable nutritional comparison across bamboo species and establish *Ch. utilis* as a premium nutritional resource, seed samples from three additional bamboo species were collected and analyzed using the same protocols as *Ch. utilis*. *Chimonobambusa tumidissinoda* seeds were collected in mid-April from Daguang County, Zhaotun City, Yunnan Province (27°49′31.11″ N, 103°46′46.20″ E), while *Sasaella kogasensis* ‘Aureostriatus’ and *Pleioblastus pygmaeus* seeds were harvested in late May from the campus of Nanjing Forestry University (32°04′40″ N, 118°48′42″ E). Collection timing was synchronized with each species’ optimal fruiting phenophase to ensure seed maturity and minimize developmental variation in nutritional composition. These three species were selected as comparative references because they represent different bamboo seed morphological types (standard caryopses, nut-like caryopses, and berry-like caryopses) and are commonly cultivated in China, thereby providing ecologically and economically relevant comparison points for *Ch. utilis*. Nutritional components analyzed included crude protein, crude fat, amino acids, vitamins, and fatty acid composition following Chinese National Food Safety Standards (GB 5009.5-2016 [39], GB 5009.6-2016 [40], GB 5009.124-2016 [41], GB 5009.82-2016 [42], and GB 5009.168-2016 [43]). All seed samples were analyzed using identical protocols through the same testing facility (Jiangsu Province Testing Center for Analysis and Measurement, Nanjing, China) to ensure inter-species analytical consistency and comparability of results.

### 2.3. Phytohormone Preparation and Seed Treatment Procedures

Three plant growth regulators were evaluated: gibberellic acid (GA_3_), 6-benzylaminopurine (6-BA), and α-naphthaleneacetic acid (NAA). Stock solutions were prepared as follows: GA_3_ (Sigma-Aldrich, St. Louis, MA, USA) was dissolved in a minimal volume of 95% ethanol (approximately 0.5 mL per 100 mL final volume) before dilution to final concentrations with ultrapure water. 6-BA (Sigma-Aldrich, St. Louis, MA, USA) was dissolved in 1 M HCl with gentle heating (approximately 0.3 mL per 100 mL final volume), then diluted with ultrapure water and pH adjusted to 5.8 using 1 M NaOH. NAA (Sigma-Aldrich, St. Louis, MA, USA) was dissolved in a minimal volume of 95% ethanol (approximately 0.5 mL per 100 mL final volume) before dilution with ultrapure water. All working solutions were freshly prepared on the day of treatment.

Three concentration levels were established for each regulator: GA_3_ at 8.66, 14.4, and 28.9 µM (equivalent to 3, 5, and 10 mg/L; molecular weight 346.37 g/mol); 6-BA at 44.4, 133.2, and 222.0 µM (equivalent to 10, 30, and 50 mg/L; molecular weight 225.25 g/mol); and NAA at 134.2, 268.5, and 402.7 µM (equivalent to 25, 50, and 75 mg/L; molecular weight 186.21 g/mol) (Supplementary Table S1). The control group received treatment with ultrapure water containing the same volume of solvent (ethanol 0.5% *v*/*v* for GA_3_ and NAA treatments; HCl-NaOH buffer for 6-BA treatment) to account for potential solvent effects on germination. Seeds were soaked in treatment solutions (1:5, seed:solution *v*/*v*) for 12 h at 22 °C in a controlled environment chamber.

Seed selection and treatment: Fresh seeds with intact seed coats (moisture content 45–50%, determined by oven-drying method at 105 °C for 24 h) were used immediately after collection without artificial drying. Following removal of debris and lemma, morphologically uniform and healthy seeds were selected and rinsed thoroughly with running water for 1 to 2 h, then rinsed again with ultrapure water. Seeds were subsequently sterilized by sequential treatment: immersion in 80% anhydrous ethanol for 1 min, followed by incubation in 0.1% potassium permanganate solution (Sinopharm Chemical Reagent Co., Ltd., Shanghai, China) for 7 min. Potassium permanganate residue was removed by thorough rinsing with ultrapure water. Seeds were subsequently transferred to germination substrates consisting of moistened sterile gauze and filter paper, which were placed in transparent culture dishes (10 cm × 10 cm × 1.5 cm) and maintained in a light incubator with controlled parameters: temperature 24 °C, photoperiod 12 h light and 12 h dark (L:D, 12:12), light intensity of 35 μmol m^−2^ s^−1^.

### 2.4. Seed Germination and Seedling Development

Seed germination and seedling development were evaluated at six predetermined developmental stages: day of PGR treatment (S1, pre-germination imbibition phase), day 4 post-treatment (S2, metabolic activation phase prior to radicle emergence), day of radicle emergence (S3, visible germination representing completion of germination sensu stricto), radicle length of 1 cm (S4, early seedling establishment phase), second true leaf expansion (S5, seedling establishment phase), and third true leaf expansion (S6, active seedling growth phase). These stages were defined to capture the continuum from seed germination (S1–S3) through seedling establishment (S4–S6). In this study, we define visible germination as the emergence of the radicle breaking through the seed coat by approximately 2 mm, which represents the morphological manifestation following the irreversible biochemical commitment to germination (chromatin remodeling and endosperm weakening).

Each treatment consisted of 600 seeds per replicate across 3 independent replicates (1800 seeds per treatment, 18,000 seeds total for ten treatments). After 12 h of PGR exposure, seeds were thoroughly rinsed with ultrapure water and placed on germination substrates. Filter paper, culture dishes, and sterile gauze were replaced daily to prevent fungal contamination. Germination data were recorded daily, including the number of radicles emerging and the timing of second and third true leaf expansion. Sampling was conducted at all six developmental stages for determination of soluble sugar and starch content as well as enzymatic activities.

The germination peak of *Ch. utilis* seeds occurred on day 7, and the germination trial continued for 30 days with daily documentation of radicle emergence and photographic records taken every other day to monitor growth and development. Germination potential (Rf, %) represents the percentage of seeds that germinate during the peak germination period and serves as an indicator of seed vigor and germination speed. It was calculated as Rf = (N_7_/Nt) × 100%, and germination percentage (Rg, %) as Rg = (N_30_/Nt) × 100%, where N_7_ is the number represents the number of normally germinated seeds within 7 days, Nt represents the total number of test seeds, and N_30_ represents the number of normally germinated seeds within 30 days. Growth parameters including root length and seedling height were measured on the day of third true leaf expansion.

### 2.5. Determination of Soluble Sugar and Starch Content

At each of the six critical developmental stages, 30 seeds per treatment were collected, rinsed with distilled water, and enzyme activity was inactivated by heated at 105 °C for 15 min. Samples were subsequently dried to constant mass at 70 °C, ground into fine powder, and precisely weighed (0.5 g). The powdered sample was suspended in 10 mL of 80% ethanol and extracted at 60 °C for 40 min, followed by centrifugation at 2146× *g* for 15 min at room temperature (Scanspeed mini centrifuge, Labogene, Denmark). The supernatant was collected and reserved. The residue was re-extracted twice with 80% ethanol, and all supernatants were combined, filtered, and diluted to 50 mL.

Soluble sugar content was quantified using the anthrone/sulphuric acid colorimetric method. Two milliliters of the extract was mixed with 8 mL of anthrone reagent (0.1% anthrone in concentrated sulphuric acid, Sinopharm Chemical Reagent Co., Ltd., Shanghai, China), heated in boiling water for 15 min, cooled to room temperature, and absorbance was measured at 620 nm using a spectrophotometer (Thermo Fisher Scientific, Waltham, MA, USA). A standard curve was established using glucose standards (0 to 100 μg/mL, Sigma-Aldrich, St. Louis, MA, USA), and soluble sugar content was expressed as a percentage of seed dry weight.

Starch content was determined indirectly as the difference between total sugar and soluble sugar content. After repeated rinsing with distilled water, the residue (0.5 g) was treated with 2 mL of 70% perchloric acid (Sigma-Aldrich, St. Louis, MA, USA) and extracted at 60 °C for 30 min, followed by centrifugation. The extraction was repeated twice with perchloric acid, supernatants were combined and diluted to 50 mL. Reducing sugars in the extract were determined using Fehling’s reagent colorimetric assay (Sinopharm Chemical Reagent Co., Ltd., Shanghai, China) at 520 nm, and starch content was calculated according to the standard curve and expressed as a percentage of seed dry weight. All assays were performed in triplicate; results are expressed as mean ± SD.

### 2.6. Enzyme Activity Assays

Sampling was conducted at the same six developmental stages as described above. For each treatment, 50 seeds were collected, rinsed with distilled water, blotted dry, and precisely weighed (0.5 g). The fresh tissue was homogenized in 5 mL of ice-cold phosphate-buffer solution (0.1 mol/L, pH 7.4, Sigma-Aldrich, St. Louis, MA, USA) containing 1% (*w*/*v*) polyvinylpyrrolidone (PVP, Sigma-Aldrich, St. Louis, MA, USA) and 1 mM dithio-threitol (DTT, Sigma-Aldrich, St. Louis, MA, USA), filtered through cheesecloth, and centrifuged at 11,200× *g* for 20 min at 4 °C. The supernatant was collected as the crude enzyme extract and stored at 4 °C for immediate analysis.

For all enzyme assays, absorbance measurements were performed using a spec-trophotometer at wavelengths specified for each enzyme. Quantification was conducted using standard curves prepared with appropriate reference compounds: glucose for sucrose synthase and starch synthase assays (Sigma-Aldrich, St. Louis, MA, USA), maltose for amylase assays (Sigma-Aldrich, St. Louis, MA, USA), and inorganic pyrophosphate for AGPase assays (Sigma-Aldrich, St. Louis, MA, USA). All enzyme activities were expressed as U/g fresh weight (U/g·FW), where one unit (U) represented the amount of enzyme catalyzing the production or consumption of 1 μmol substrate per minute. All assays were performed in triplicate with appropriate blank and positive controls to ensure accuracy and reliability. All enzyme activities were expressed on the basis of fresh tissue weight (g fresh weight, g·FW) per unit time, with specific units provided in each subsection below.

#### 2.6.1. Sucrose-Metabolizing Enzyme Activities

Invertase (INV) activity was assayed by mixing 0.5 mL of crude enzyme extract with 0.5 mL of 1% sucrose substrate (prepared in pH 7.4 phosphate buffer) and incubating at 37 °C for 60 min. The reaction was terminated by adding 0.5 mL of TBA reagent (Sinopharm Chemical Reagent Co., Ltd., Shanghai, China) and boiling for 10 min. Reducing sugar concentration was determined using the 3,5-dinitrosalicylic acid (DNS) colorimetric method at 540 nm with a glucose standard curve. Enzyme activities were expressed as μmol NADH g^−1^ h^−1^ based on fresh tissue weight (g·FW).

#### 2.6.2. Starch-Degrading Enzyme Activities

α-Amylase (α-Amy) activity was determined by incubating 0.5 mL crude enzyme extract with 4.5 mL of 1% soluble starch substrate (Sigma-Aldrich, St. Louis, MA, USA) (pH 7.4) at 37 °C for 30 min. Following the addition of 1 mL DNS reagent (Sigma-Aldrich, St. Louis, MA, USA) and boiling for 10 min, samples were cooled to room temperature and absorbance was measured at 540 nm with a maltose standard curve. Enzyme activities were expressed as mg g^−1^ min^−1^ based on fresh tissue weight (g·FW).

β-Amylase (β-Amy) activity was assayed similarly, with 0.5 mL crude enzyme extract incubated in 4.5 mL starch substrate (prepared in pH 5.5 acetate buffer) at 37 °C for 60 min. Colorimetric detection and quantification followed the same procedure as α-amylase, with pre-stopped control samples serving as controls. Enzyme activities were expressed as mg g^−1^ min^−1^ based on fresh tissue weight (g·FW).

#### 2.6.3. Starch-Synthesizing Enzyme Activities

Starch synthase (SS) activity was determined by incubating 0.5 mL crude enzyme extract in a reaction mixture consisting of ADP-glucose (100 mmol/L, 0.5 mL, Sigma-Aldrich, St. Louis, MA, USA), starch substrate (0.5 mL, pH 7.4 phosphate buffer), and calcium chloride (5 mmol/L, 0.2 mL, Sinopharm Chemical Reagent Co., Ltd., Shanghai, China) at 37 °C for 60 min. The reaction was terminated by adding perchloric acid solution, centrifuged, and the supernatant was developed with anthrone reagent (Sinopharm Chemical Reagent Co., Ltd., Shanghai, China). Absorbance was measured at 620 nm with a glucose standard curve. Enzyme activities were expressed as μmol g^−1^ h^−1^ based on fresh tissue weight (g·FW).

ADP-glucose pyrophosphorylase (AGPase) activity was assessed by incubating 0.5 mL crude enzyme extract in a reaction mixture containing glucose-1-phosphate (50 mmol/L, Sigma-Aldrich, St. Louis, MA, USA), ATP (10 mmol/L, Sigma-Aldrich, St. Louis, MA, USA), magnesium chloride (10 mmol/L, Sinopharm Chemical Reagent Co., Ltd., Shanghai, China), and phosphate buffer (pH 7.4, Sigma-Aldrich, St. Louis, MA, USA) at 37 °C for 30 min. The reaction was terminated and inorganic pyrophosphate content was quantified at 690 nm using molybdenum/reducing agent reagent colorimetry (Sinopharm Chemical Reagent Co., Ltd., Shanghai, China). Enzyme activities were expressed as μmol g^−1^ h^−1^ based on fresh tissue weight (g·FW). GBSS activity was determined using the same methodology and expressed as μmol g^−1^ h^−1^ based on fresh tissue weight (g·FW).

### 2.7. Statistical Analysis

Data were collated using Microsoft Excel 2022 (Microsoft Corporation, Redmond, WA, USA) and IBM SPSS 22.0 (IBM Corporation, Armonk, NY, USA). One-way analysis of variance (ANOVA) with post hoc tests (LSD and Duncan) was performed using IBM SPSS 22.0 to assess significant differences in soluble sugar, starch, and enzyme activity among across concentration levels at various developmental stages (significance threshold: *p* < 0.05).

## 3. Results

### 3.1. Nutritional Quality Assessment of Seeds in Ch. utilis

**Figure 1:**
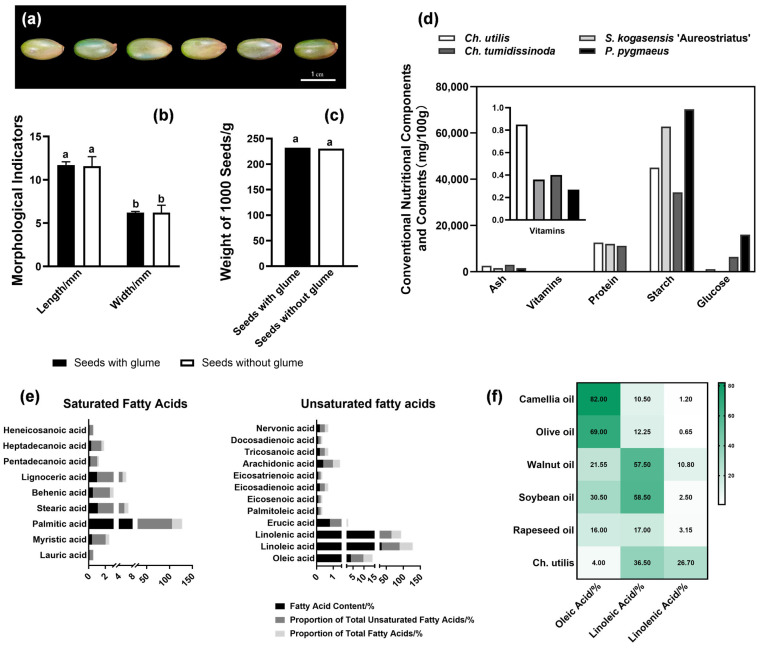
Morphological characteristics and conventional nutrient contents of *Ch. utilis* seeds. (**a**) Seed morphology of *Ch. utilis*. (**b**,**c**) Morphological parameters of *Ch. utilis* seeds. (**d**) Conventional nutritional components and contents of *Ch. utilis* seeds. (**e**) Content of saturated fatty acids and unsaturated fatty acids in seeds of *Ch. utilis*. (**f**) Comparison of unsaturated fatty acid content between *Ch. utilis* seeds and common edible oils. Note: Different letters in the graph indicate significant differences at a level of *p* < 0.05.

Seeds of *Ch. utilis* were ellipsoidal, berry-like caryopses (Figure 1a). After removal of the glumes, seeds measured 11.58 ± 1.09 mm in length and 6.20 ± 0.87 mm in diameter, with a thousand-seed weight of 230.3 ± 0.0014 g (Figure 1b,c). Nutritional composition analysis revealed that, compared to other bamboo species including *Ch*. *tumidissinoda*, *Sk* ‘Aureostriatus’, and *P*. *pygmaeus*, *Ch. utilis* seeds exhibited the highest vitamin content (0.85 mg/100 g), exceeded other bamboo species by 1.5- to 2.0-fold. Ash content ranked second highest (2600 mg/100 g), indicating substantial mineral accumulation capacity (Figure 1d). The amino acid profile was complete, comprising 18 species, wherein hydrolyzed amino acids (8.02%) were 7.9 times higher than free amino acids (1.01%) (Supplementary Figure S1). Non-essential amino acids comprised 73.69% of total amino acids, with essential amino acids constituting 26.31%. Aspartic acid and glutamic acid predominated, accounting for 28.7% and 17.72% of total amino acids, respectively. Both are hydrolyzed, non-essential amino acids with significant nutritional and functional value. The fatty acid composition comprised 9 saturated and 12 unsaturated fatty acids (Figure 1e). Among saturated fatty acids, palmitic acid was most abundant (20.7%), representing 84.84% of total saturated fatty acids. Unsaturated fatty acids constituted 67.39% of total lipids, predominantly linoleic acid (36.5%) and linolenic acid (26.7%), which accounted for 54.16% and 39.62% of total unsaturated fatty acids, respectively. Compared to five major vegetable oils (camellia, olive, rapeseed, peanut, and walnut oils), *Ch. utilis* seeds contained 1.5 to 3.3 times higher linoleic acid content than rapeseed oil and 2.1 to 2.8 times higher linolenic acid content than walnut oil, demonstrating potential as a premium edible oil source (Figure 1f).

### 3.2. Regulatory Effects of Plant Growth Regulators on Seed Germination

**Figure 2:**
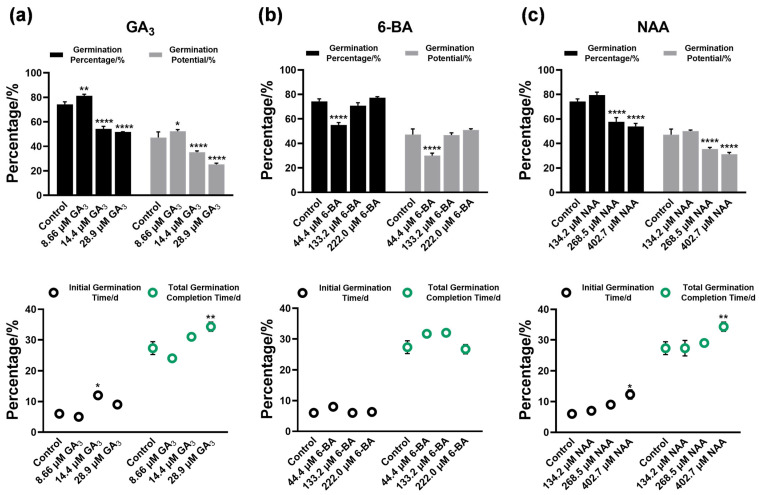
Effects of plant growth regulators at different concentrations on seed germination of *Ch. utilis*. (**a**–**c**) Comparative analysis of seed germination parameters including germination percentage, germination potential, time to initial germination, and time to complete germination in response to different concentrations of GA_3_ (**a**), 6-BA (**b**), and NAA (**c**) relative to the control treatment. Asterisks indicate significant differences from control based on Dunnett’s test: **** *p* < 0.0001, ** *p* < 0.01, * *p* < 0.05. Data represent means from three biological replicates.

All three PGRs exhibited concentration-dependent regulation of seed germination with distinct response patterns (Figure 2). GA_3_ displayed typical biphasic regulation, promoting germination at low concentrations while inhibiting at high concentrations (Figure 2a). Treatment with 8.66 µM GA_3_ yielded the highest promotive effects, significantly increasing germination percentage and germination potential by 6.3% and 6.65% relative to controls, respectively, reducing time to initial germination to 5 days, and advancing the germination cycle by 5 days. Conversely, 28.9 µM GA_3_ significantly suppressed germination, decreasing germination percentage and energy by 23.3% and 21.7%, respectively, with initial germination delayed by 3 to 5 days (*p* < 0.05).

6-BA displayed nonlinear concentration-response kinetics, with low concentrations exhibiting weaker promotive capacity compared to intermediate and high concentrations, while intermediate and high concentrations showing substantially enhanced promotion of germination (Figure 2b). Promotive effects progressively intensified with increasing concentration. At 222.0 µM, germination percentage peaked at 77.5% (1.03-fold increase relative to control), germination potential reached 50.8%, time to initial germination advanced to 6 days (1 day earlier than control), with the shortest total germination duration. At 44.4 µM, germination potential was lowest at 30% (16.7% below control), indicating significant inhibitory effects at low 6-BA concentrations (*p* < 0.05).

NAA exhibited unimodal dose–response kinetics with a distinct concentration-response pattern (Figure 2c). At 134.2 µM, the highest promotive effects were observed, achieving 79.20% germination percentage (4.2% increase relative to control) and 50% germination potential (3.3% increase vs. control), with initial germination at 7 days (2 days earlier than control) and shortest completion time. As concentration increased to 268.5 to 402.7 µM, both germination percentage (57.5% to 53.3%) and energy (35.8% to 31.6%) declined substantially, demonstrating a narrow concentration range for NAA-mediated germination promotion (*p* < 0.05). These results demonstrate that all three PGRs effectively reduced the germination duration of *Ch. utilis* seeds, with NAA and 6-BA treatments at 134.2 to 222.0 µM exhibiting the most pronounced efficacy.

### 3.3. Phytohormone Regulation of Seed Storage Substance Dynamics in Ch. utilis Seeds

**Figure 3:**
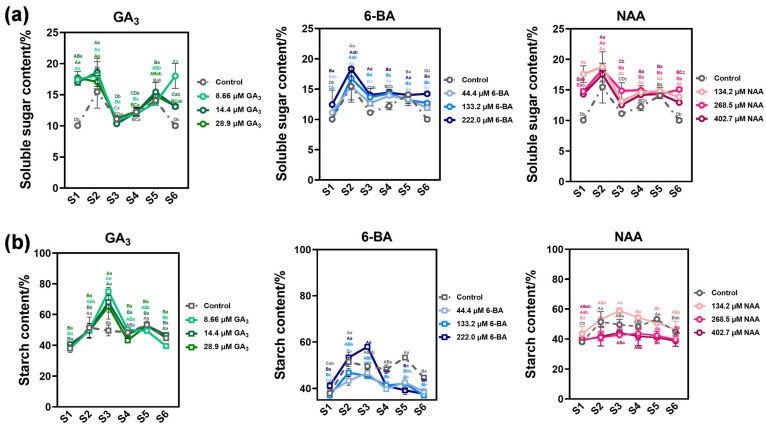
Dynamic changes in storage substances of *Ch. utilis* seeds. Changes in soluble sugar (**a**) and starch (**b**) contents of *Ch. utilis* seeds under different concentrations of GA_3_, 6-BA, and NAA treatments. Data are presented as means ± standard error (n = 3). Different uppercase letters indicate significant differences among treatments at the same germination stage (*p* < 0.05, one-way ANOVA followed by Duncan’s multiple range test). Different lowercase letters indicate significant differences among germination stages within the same treatment (*p* < 0.05). Note: Different letters in the graph indicate significant differences at a level of *p* < 0.05.

Soluble sugar content exhibited biphasic temporal dynamics during germination, initially increasing then declining, with the most consumption occurring prior to radicle emergence (S2 stage). Exogenous application of GA_3_ (8.66 µM), 6-BA (222.0 µM), and NAA (134.2 µM) maintained elevated soluble sugar concentrations during early germination stages (S1–S3) (*p* < 0.05), which likely supports both metabolic energy demands and molecular processes including chromatin remodeling, DNA synthesis, and de novo protein synthesis that characterize the germination sensu stricto phase. These elevated sugar levels may function not only as respiratory substrates but also as signaling molecules that coordinate germination-associated gene expression (Figure 3a). Starch content displayed a triphasic pattern comprising accumulation, plateau, and mobilization phases, peaking at S3 (Figure 3b). All three PGRs significantly promoted both starch accumulation and subsequent remobilization, with NAA (134.2 µM) and 6-BA (222.0 µM) inducing the most pronounced responses.

**Figure 4:**
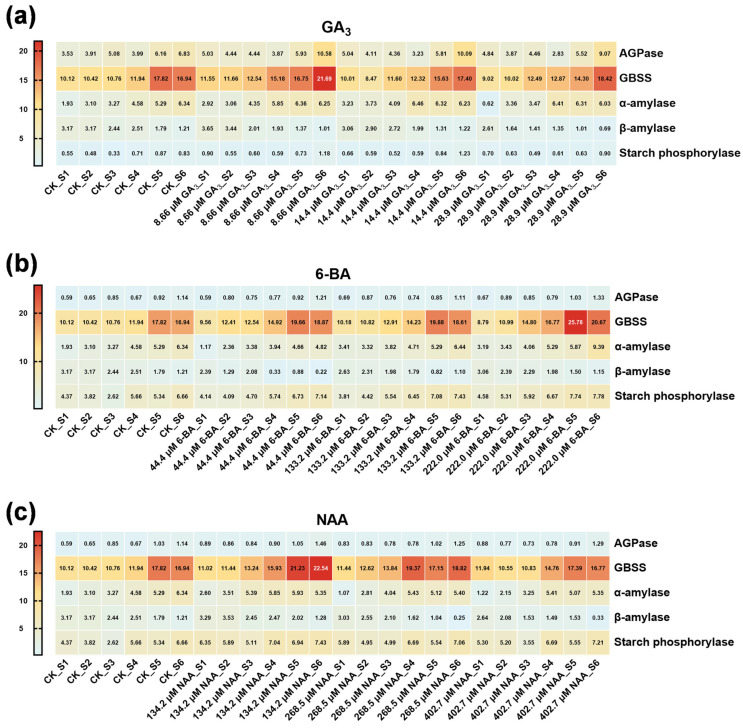
Heatmap of starch metabolism enzyme activities in seeds of *Ch. utilis* under different treatments. Activities of AGPase, GBSS, α-amylase, β-amylase, and starch phosphorylase under different concentrations of GA_3_ (**a**), 6-BA (**b**), and NAA (**c**) treatments. Enzyme activities for AGPase and GBSS are expressed as μmol g^−1^ h^−1^ (based on fresh tissue weight, g·FW), while α-amylase, β-amylase, and starch phosphorylase are expressed as mg g^−1^ min^−1^ (based on fresh tissue weight). The numerical value at the center of each cell indicates the specific enzyme activity, while the color intensity represents the magnitude of enzymatic activity, with darker colors corresponding to higher activity levels. Each enzyme’s activity profile reveals distinct responses to varying phytohormone concentrations, providing insights into the hormonal regulation of starch metabolism during seed development.

The three hormones exerted differential modulatory effects on starch-metabolizing enzymes (Figure 4 and Supplementary Table S2). All enzyme activities are expressed as μmol g^−1^ h^−1^ based on fresh tissue weight (g·FW), unless otherwise specified. GA_3_ (8.66 µM) treatment progressively enhanced granule-bound starch synthase (GBSS) activity, reaching maximum levels at S6 (*p* < 0.05 versus controls) (Figure 4a). 6-BA (222.0 µM) significantly upregulated both ADP-glucose pyrophosphorylase (AGPase) and GBSS activities throughout S2–S6 (*p* < 0.05), demonstrating stage-specific modulatory patterns. (Figure 4b). NAA (134.2 µM) most robustly stimulated AGPase and GBSS activities, sustaining elevated enzymatic activity throughout the germination process (Figure 4c). Regarding starch catabolism, all three hormones enhanced α-amylase and starch phosphorylase activities, whereas β-amylase activity progressively declined.

**Figure 5:**
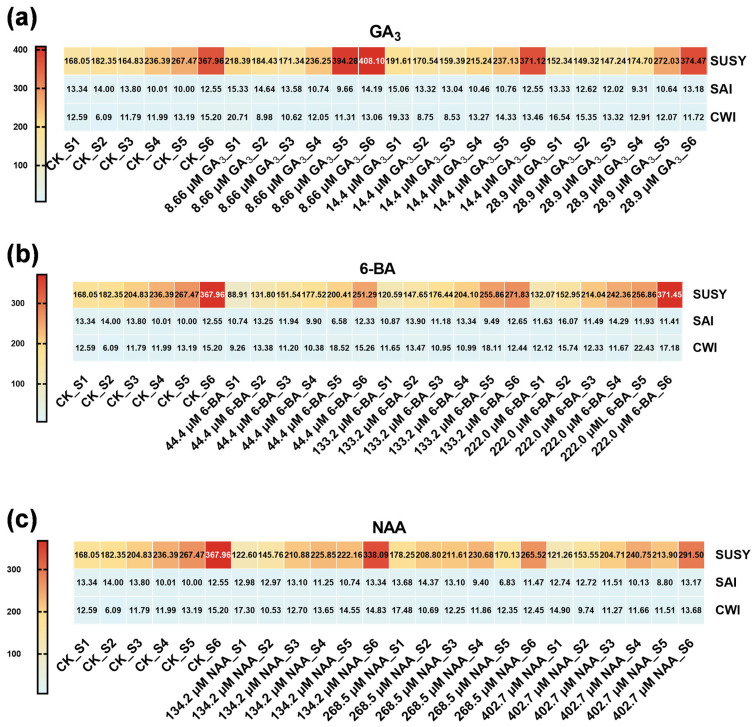
Heatmap analysis of sucrose-metabolizing enzyme activities in *Ch. utilis* seeds under different concentrations of plant growth regulators. Enzyme activities of SUSY, SAI, and CWI in response to varying concentrations of GA_3_ (**a**), 6-BA (**b**), and NAA (**c**) treatments. All enzyme activities are expressed as μmol NADH g^−1^ h^−1^ based on fresh tissue weight (g·FW). Values within each cell represent specific enzyme activities (unit specification should be added if not in the figure), with color intensity indicating the magnitude of enzymatic activity (darker colors correspond to higher activity levels).

Sucrose metabolism represented a central regulatory node in phytohormone-mediated seed germination, with the three major sucrose-metabolizing enzymes displaying distinctive hormone-responsive patterns (Figure 5 and Supplementary Table S2). All sucrose-metabolizing enzyme activities (SUSY, SAI, and CWI) are expressed as μmol NADH g^−1^ h^−1^ based on fresh tissue weight (g·FW). Treatment with 6-BA suppressed sucrose synthase (SUSY) activity throughout the germination period (*p* < 0.05), and this inhibitory effect attenuated progressively with increasing hormone concentration (Figure 5b). High-concentration NAA (268.5 to 402.7 µM) induced concentration-dependent suppression of SUSY activity (*p* < 0.05) (Figure 5c), whereas GA_3_ treatment produced minimal modulatory effects on SUSY (Figure 5a). In contrast, sucrose invertase (SAI) and cell wall invertase (CWI) exhibited biphasic temporal dynamics: both enzymes declined through mid-germination stages (SAI minimal at S4; CWI minimal at S2, *p* < 0.05) and subsequently recovered during late germination, while SUSY activity increased progressively throughout the germination period. These hormone-specific responses suggest that differential modulation of sucrose utilization enzymes orchestrates developmental transitions during seed germination.

### 3.4. Effects of PGR on Growth Traits of Ch. utilis Seedling

**Figure 6:**
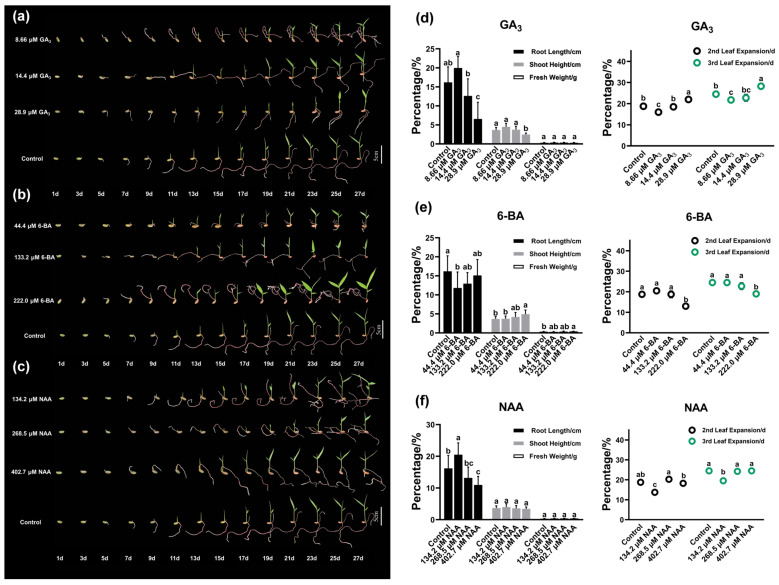
Effects of three PGRs on *Ch. utilis* seedling growth. (**a**–**c**) Effects of different concentrations of GA_3_ (**a**), 6-BA (**b**), and NAA (**c**) on plant height, primary root length, lateral root number, and leaf expansion time of *Ch. utilis*. (**d**–**f**) Comprehensive comparison of growth parameters under different concentrations of GA_3_ (**d**), 6-BA (**e**), and NAA (**f**) treatments. Bar chart data are presented as mean ± SD (n ≥ 3). Note: different letters indicate significant differences (*p* < 0.05).

GA_3_ exhibited a pronounced concentration-dependent threshold effect on seedling growth parameters (Figure 6a,d). At 8.66 µM, root length and shoot height reached 19.96 ± 3.03 cm and 4.49 ± 0.93 cm, respectively, which were significantly superior to the controls (*p* < 0.05). However, 28.9 µM GA_3_ significantly inhibited both parameters, reducing them to 6.57 ± 4.4 cm and 2.48 ± 0.39 cm (*p* < 0.05), with correspondingly delayed leaf expansion, thereby indicating a defined promotional threshold for GA_3_ effects on seedling growth in this bamboo species.

6-BA treatment demonstrated concentration-dependent promotional effects across the tested range (Figure 6b,e). With increasing concentrations (44.4 to 222.0 µM), seedling height and root length increased progressively, lateral root numbers increased, and leaf expansion time shortened (Figure 6b). Treatment with 222.0 µM proved most effective, producing a seedling height of 4.92 ± 1.08 cm (*p* < 0.05), with leaf expansion occurring 5 days earlier than the controls (second and third leaves expanded at 13 ± 0.82 d and 19 ± 0.82 d, respectively), and a fresh weight of 0.39 ± 0.07 g (Figure 6e). A comprehensive assessment of root system architecture revealed that while primary root elongation was reduced, the substantial increase in lateral root proliferation resulted in significantly enhanced total root exploration surface area and overall root biomass accumulation, as evidenced by the increased seedling fresh weight. This shift from primary root dominance toward lateral root-based architecture represents an adaptive response to 6-BA treatment, enhancing the overall root system functional capacity for resource acquisition.

NAA treatment displayed an asymmetric “low-concentration promotion, high-concentration inhibition” response pattern (Figure 6c,f). At 134.2 µM, root length (20.51 ± 3.72 cm) and shoot height (3.96 ± 1.07 cm) increased by 27% and 8% relative to the controls, respectively, with leaf expansion occurring 5 days earlier (expansion times at 13.75 ± 0.96 d and 19.5 ± 1.29 d, respectively), representing the strongest root-promoting effect among the three hormones tested. All NAA treatments significantly enhanced seedling fresh weight (*p* < 0.05), suggesting universal promotional effects on seedling biomass accumulation.

### 3.5. Multifactorial Comprehensive Evaluation

**Figure 7:**
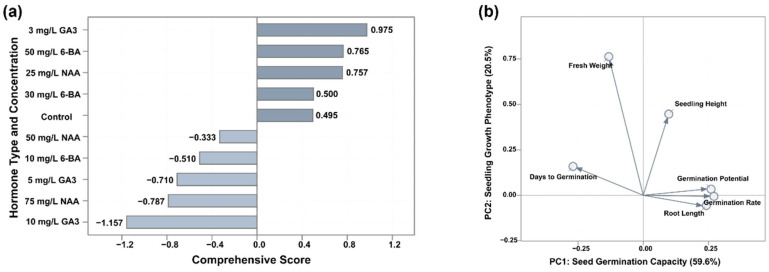

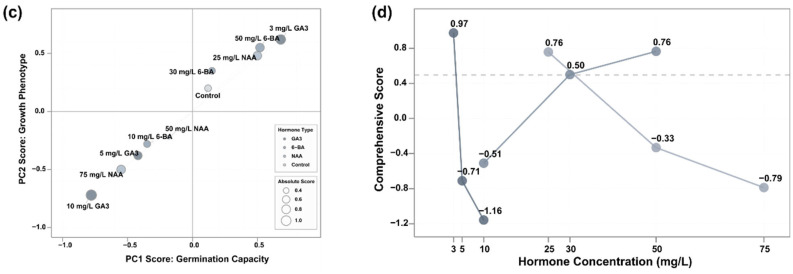
Multi-dimensional assessment of factor analysis on seed germination and seedling growth of *Ch. utilis*. (**a**) Comprehensive score ranking bar chart of 10 treatments, with darker and lighter gray bars representing positive promotion and negative inhibition effects, respectively. Comprehensive scores were calculated by weighted summation: SCORE = 0.744 × PC1 + 0.256 × PC2. (**b**) Biplot of factor loadings, clearly presenting the indicator composition of seed germination capacity factor (PC1, 59.57%) and seedling growth vigor factor (PC2, 20.54%). (**c**) Multivariate space scatter plot showing the distribution of 10 treatments in the two-principal component coordinate system. (**d**) Concentration-response curves of three hormones, with GA_3_, 6-BA, and NAA represented by different shades of gray.

Factor analysis based on six key indicators of seed germination and seedling growth (root length, germination percentage, germination potential, shoot height, fresh weight, and initial germination time) extracted two common factors with a cumulative variance contribution of 80.11%. The first common factor (accounting for 59.57% of the variance) was composed of root length, germination percentage, and germination potential, and primarily represented seed germination capacity. The second common factor (accounting for 20.54% of the variance) was composed of shoot height, fresh weight, and initial germination time, and primarily represented seedling growth potential (Figure 7). The biplot of factor loadings clearly shows the indicator composition of these two factors (Figure 7b), while the multivariate scatter plot shows the distribution patterns of all treatments in the two-dimensional principal component space (Figure 7c). Comprehensive scores were calculated for all treatments and ranked (Figure 7a), with positive scores indicating promotion and negative scores indicating inhibition. These optimal treatments clustered in quadrant I, demonstrating superior performance in both germination capacity and seedling vigor. In contrast, suboptimal treatments were clustered in quadrant III, whereas the control group was located near the origin (Figure 7c). All hormone concentration-response curves display characteristic single-peak patterns, indicating the existence of clearly defined optimal concentrations (Figure 7d). Comprehensive evaluation identified the optimal treatment concentrations for *Ch. utilis* seeds as: GA_3_ at 8.66 µM, 6-BA at 222.0 µM, and NAA at 134.2 µM.

## 4. Discussion

### 4.1. Nutritional Advantages and Edible Potential of Ch. utilis Seeds

Comprehensive nutritional analysis revealed that *Ch. utilis* seeds possess exceptional nutritional quality, particularly high unsaturated fatty acid content (67.39% of total lipids), complete amino acid profile, and elevated micronutrient levels. The unsaturated fatty acid composition is dominated by linoleic acid (36.5%) and α-linolenic acid (26.7%), positioning *Ch. utilis* seeds as a candidate for functional food development. The omega-6/omega-3 ratio, primarily determined by linoleic and linolenic acid composition, plays a pivotal role in regulating inflammatory responses and cardiovascular health [44]. The linolenic acid content exceeds that of walnut oil by 2.1- to 2.8-fold. As a precursor to DHA, linolenic acid exhibits established benefits in lipid regulation and anti-inflammatory responses [45]. Given the high linolenic acid content and balanced polyunsaturated fatty acid profile of *Ch. utilis* seeds, this species demonstrates nutritional potential comparable to traditionally recognized healthy oils such as flaxseed and perilla oils, positioning it as a candidate material for functional edible oil development.

The amino acid composition features high proportions of aspartic acid (28.7%) and glutamic acid (17.72%). These amino acids function both as metabolic substrates and signaling molecules during germination [46,47]. Glutamic acid participates in nitrogen remobilization and serves as a precursor for γ-aminobutyric acid (GABA) biosynthesis [48]. The 7.9-fold enrichment of protein-bound relative to free amino acids indicates well-structured storage proteins that may enhance bioavailability [49]. These nutritional attributes position *Ch. utilis* seeds as a high-value resource for both edible oil extraction and protein-based functional food development.

### 4.2. Differential Phytohormone Regulation Patterns and Physiological Mechanisms

Plant hormones orchestrate seed germination through complex signaling networks that integrate environmental cues with endogenous developmental programs [50,51]. Our findings reveal distinct concentration-response kinetics for GA_3_, 6-BA, and NAA in *Ch. utilis* seed germination. The biphasic response to GA_3_ (promotion at 8.66 µM, inhibition at 28.9 µM) aligns with established GA signaling paradigms. Optimal GA levels promote germination through a spatially and temporally coordinated process involving DELLA protein degradation that occurs differentially across seed tissues. In the embryo, GA-mediated DELLA degradation activates cell division and elongation programs, while in the aleurone layer, it triggers the expression of hydrolytic enzymes including α-amylase that mobilize endosperm reserves. This tissue-specific signaling creates auxin and GA gradients that coordinate embryonic axis growth with reserve mobilization [52]. Supraoptimal GA concentrations may trigger feedback inhibition or interfere with GA-ABA antagonism, thereby disrupting the hormonal balance required for coordinated germination [53]. This biphasic pattern emphasizes the requirement for GA homeostasis maintenance during germination.

The nonlinear 6-BA response (suboptimal response at low concentration (44.4 µM) transitioning to strong promotion at high concentrations (222.0 µM)) reflects species-specific concentration-dependent patterns of cytokinin signaling, potentially involving differential activation of type-A versus type-B ARABIDOPSIS RESPONSE REGULATOR (ARR) pathways or differential sensitivity of downstream developmental programs to cytokinin levels [54]. This concentration-dependent pattern is consistent with cytokinin’s known biphasic effects, where suboptimal concentrations may be insufficient to activate robust developmental responses, while higher concentrations achieving the concentration threshold required for promoting seed germination and seedling development in *Ch utilis*. The preferential stimulation of lateral root proliferation concurrent with reduced primary root elongation under all 6-BA concentrations reflects a well-characterized cytokinin-mediated architectural remodeling of root systems, which involves modulation of auxin transport and local auxin maxima, consistent with established models of lateral root initiation [55,56]. Despite the reduction in primary root length, the enhanced lateral root formation results in a net increase in total root system length and surface area, thereby enhancing overall nutrient and water uptake capacity. This response pattern bears functional similarity to cytokinin regulation in *Arabidopsis*, where exogenous cytokinin application modulates primary root meristem activity while promoting lateral root founder cell specification, creating a more branched and explorative root architecture optimal for seedling establishment [57].

NAA exhibited classical auxin dose–response kinetics with a clear hormetic window. At the optimal concentration (134.2 µM), exogenous NAA functions as a physiological complement to endogenous auxin, maintaining appropriate auxin gradients and establishing proper auxin maxima that are essential for coordinated root initiation, meristem maintenance, and lateral root founder cell specification. The superior root-promoting efficacy of NAA (27% increase in root length) compared to 6-BA and GA_3_ underscores the primacy of auxin signaling in root system architecture determination [58]. However, it is important to note that enhanced root length during early seedling establishment (prior to full photosynthetic competence) represents mobilization of seed endogenous reserves rather than net biomass gain. The benefit of early vigorous root growth lies in establishing optimal root system architecture for subsequent resource acquisition once photosynthesis commences. The significantly increased seedling fresh weight across all NAA treatments (*p* < 0.05) suggests that the enhanced root development does not excessively deplete seed reserves, and the improved resource acquisition capacity ultimately compensates for initial reserve investment.

In contrast, at higher NAA concentrations (268.5 to 402.7 µM), the excessive accumulation of exogenous auxin disrupts the endogenous auxin gradient by overwhelming intrinsic auxin homeostasis mechanisms. This supraoptimal auxin accumulation disturbs the spatial distribution of auxin maxima required for coordinated developmental processes, leading to auxin-induced loss of meristem identity, ectopic auxin signaling, and subsequent growth arrest. The pronounced growth inhibition at these high concentrations likely involves auxin-gradient-dependent disruption of auxin signaling output, potentially through auxin-induced ethylene biosynthesis and subsequent growth arrest [59].

An important consideration regarding the relatively high phytohormone concentrations required for *Ch. utilis* seed germination concerns the potential penetration barriers imposed by seed anatomical structure. Unlike model species with thin, permeable seed coats, *Ch. utilis* possesses berry-like caryopses with fleshy pericarp and thick seed coats that may substantially impede hormone uptake. The high external treatment concentrations (8.66–28.9 µM GA_3_, 44.4–222.0 µM 6-BA, 134.2–402.7 µM NAA) may therefore be necessary to establish sufficient concentration gradients for passive diffusion across multiple tissue layers, rather than reflecting inherently low hormone sensitivity. Quantitative determination of endogenous hormone levels in seed tissues following exogenous treatment would provide direct evidence for this penetration barrier hypothesis and clarify the relationship between applied concentrations and bioavailable concentrations at target sites. Such analyses represent an important direction for future mechanistic investigation. Nevertheless, the present physiological and biochemical data demonstrate that the tested concentration ranges effectively promote germination and seedling establishment, providing practical protocols for *Ch. utilis* propagation optimization.

### 4.3. Enzymatic Regulation of Starch and Sucrose Metabolism

Changes in storage substance content reflected material translocation patterns during seed germination processes, with alterations in stored reserves representing crucial physiological transformations that broke seed dormancy and promoted germination [60,61,62]. For bamboo seeds, including *Dendrocalamus brandisii* and *Phyllostachys edulis*, soluble sugar content during germination displayed patterns of initial increase followed by decline. In the present study, control treatment variations aligned with these documented bamboo soluble sugar content change trends [63]. Soluble sugars accumulate during early germination (S1–S3) with most consumption occurring before radicle emergence. Exogenous hormone treatments extended the maintenance period of elevated soluble sugar concentrations, providing more abundant carbon source supply [64]. Starch content exhibited three distinct phases: an accumulation phase (S1–S2), followed by a plateau phase (S3), and subsequently a mobilization phase (S4–S6), with peak levels observed at S3 coinciding with radicle emergence. This pattern corresponds to critical transitions in starch metabolism, suggesting that radicle emergence represents a pivotal milestone in the internal metabolic state of seeds. All three hormones significantly promoted both starch accumulation and subsequent mobilization, with NAA and 6-BA showing the most pronounced effects, consistent with their strong activation of AGPase and GBSS [65].

Enzymes functioned as biological catalysts with crucial roles in material metabolism, morphological development, and energy transformation within plant systems [66]. During seed germination, enzymes catalyzed and regulated substance transformations, converting materials into transportable forms to supply energy for growth and synthetic reactions [67]. In starch biosynthesis pathways, AGPase transferred glucose residues from glucose-1-phosphate to ATP, generating ADP-glucose for subsequent starch synthesis, representing a critical regulatory site and rate-limiting enzyme in starch synthesis [68]. GBSS promoted starch granule synthesis and aggregation, with these stored starch granules providing energy substrates required for germination processes. GBSS was exclusively involved in amylose synthesis, catalyzing extension of long glucan chains [69]. In the present study, seeds treated with 6-BA, NAA, and GA_3_ exhibited elevated starch synthase activities compared to controls, with enhanced activities further promoting starch synthesis, signifying intensified starch granule synthesis and aggregation processes.

Regarding starch catabolism, all three hormones enhanced α-amylase and starch phosphorylase activities, while β-amylase activity progressively declined. α-Amylase and β-amylase constitute the two principal enzymes responsible for starch degradation during seed germination, with α-amylase playing primary roles in native starch granule degradation through expression regulated by both gibberellin and sugar demand/starvation [70]. This differentiated enzyme activity pattern suggests that α-amylase and starch phosphorylase are primary participants in starch mobilization, while β-amylase likely plays a secondary role.

Sucrose metabolism represents a pivotal regulatory node integrating carbon partitioning with developmental progression [71]. The progressive elevation of SUSY activity observed throughout germination and early seedling establishment aligns with previous reports in *Arabidopsis* [72]. SUSY supplies UDP-glucose for cellulose biosynthesis and hexose phosphates for glycolytic pathways. This functional versatility may explain why SUSY mutants exhibit severe developmental defects during germination, as disruption of this enzyme simultaneously impairs both structural and energetic requirements for seedling establishment [73,74,75]. The concentration-dependent inhibition by 6-BA and supraoptimal NAA, contrasting with GA_3_’s negligible effects, suggests that cytokinins and auxins may directly antagonize sucrose catabolism to redirect carbon flux toward storage or alternative metabolic pathways.

## 5. Conclusions

In conclusion, seeds of *Ch*. *utilis* exhibited exceptional nutritional quality, characterized by outstanding vitamin content, complete amino acid composition, and optimal unsaturated fatty acid profiles, demonstrating significant potential for edible oil development. To achieve optimal nutritional value and ensure successful propagation of this crop, effective management of seed germination and seedling growth is essential. Our findings demonstrate that PGR treatments precisely regulated seed germination and seedling growth through differential modulation of carbohydrate metabolism, a critical physiological process underlying nutritional accumulation and seedling vigor. Notably, sucrose metabolic enzymes, particularly sucrose synthase (SUSY), displayed the most sensitive hormonal responses with pronounced hormone-type specificity, representing a pivotal regulatory target in the carbohydrate metabolism-related germination control mechanism. Although the three PGRs operated through distinct mechanisms, all exhibited clear concentration-threshold effects whereby promotional effects diminished or reversed to inhibition beyond optimal concentrations (GA_3_: 8.66 µM; 6-BA: 222.0 µM; NAA: 134.2 µM). GA_3_ and NAA functioned through bidirectional regulatory mechanisms, whereas 6-BA displayed unidirectional progressive promotion. These findings provide crucial theoretical guidance and technical support for optimizing seed germination protocols, seedling cultivation practices, and industrial-scale propagation of *Ch. utilis*.

## Data Availability

The original contributions presented in this study are included in the article/Supplementary Materials. Further inquiries can be directed to the corresponding author.

## Supplementary Materials

The following supporting information can be downloaded at: https://www.mdpi.com/article/10.3390/plants14243780/s1, Figure S1: Amino acid contents in *Ch. utilis* seeds. (a) Contents of hydrolyzed amino acids and their percentages of total amino acids. (d) Contents of free amino acids and their percentages of total amino acids. Note: * represents essential amino acids; Figure S2: Effects of different concentrations of GA_3_, 6-BA, and NAA on the accumulation of storage substances in *Ch. utilis* seeds at different developmental stages. Figure a represents the soluble sugar powder content; Figure b represents the starch content. Note: Different capital letters indicate significant differences between the same concentration in different periods, and different lowercase letters indicate significant differences between different concentrations in the same period (*p* < 0.05); Table S1: Experimental design for seed treatment of *Chimonobambusa utilis* with plant growth regulators at different concentrations; Table S2: Effects of different concentrations of plant growth regulators on enzyme activities of *Ch. utilis* seeds at different stages.
