# Supplementary material for: Comprehensive Phytohormone Analysis Reveals the Roles of Auxin, Cytokinin, and Gibberellin in Enhancing Seed Germination and Growth of Chimonobambusa utilis
Source: Plants (Basel). 2025 Dec 11;14(24):3780. doi: 10.3390/plants14243780 (PMC12736858; doi:10.3390/plants14243780)
Supplement: Supplementary file 1 [file plants-14-03780-s001.zip › Supplementary Figure.pdf]

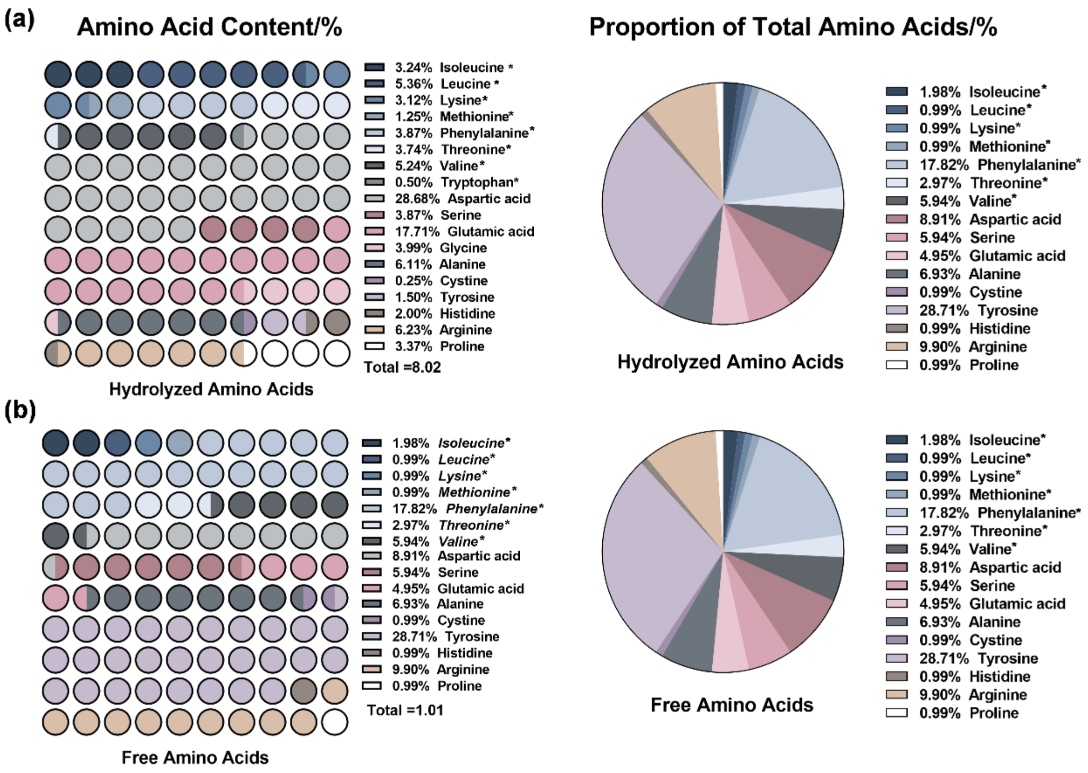

Supplementary Figure S1 Amino acid contents in *Ch. utilis* seeds. (a) Contents of hydrolyzed amino acids and their percentages of total amino acids. (d) Contents of free amino acids and their percentages of total amino acids. Note: * represents essential amino acids.

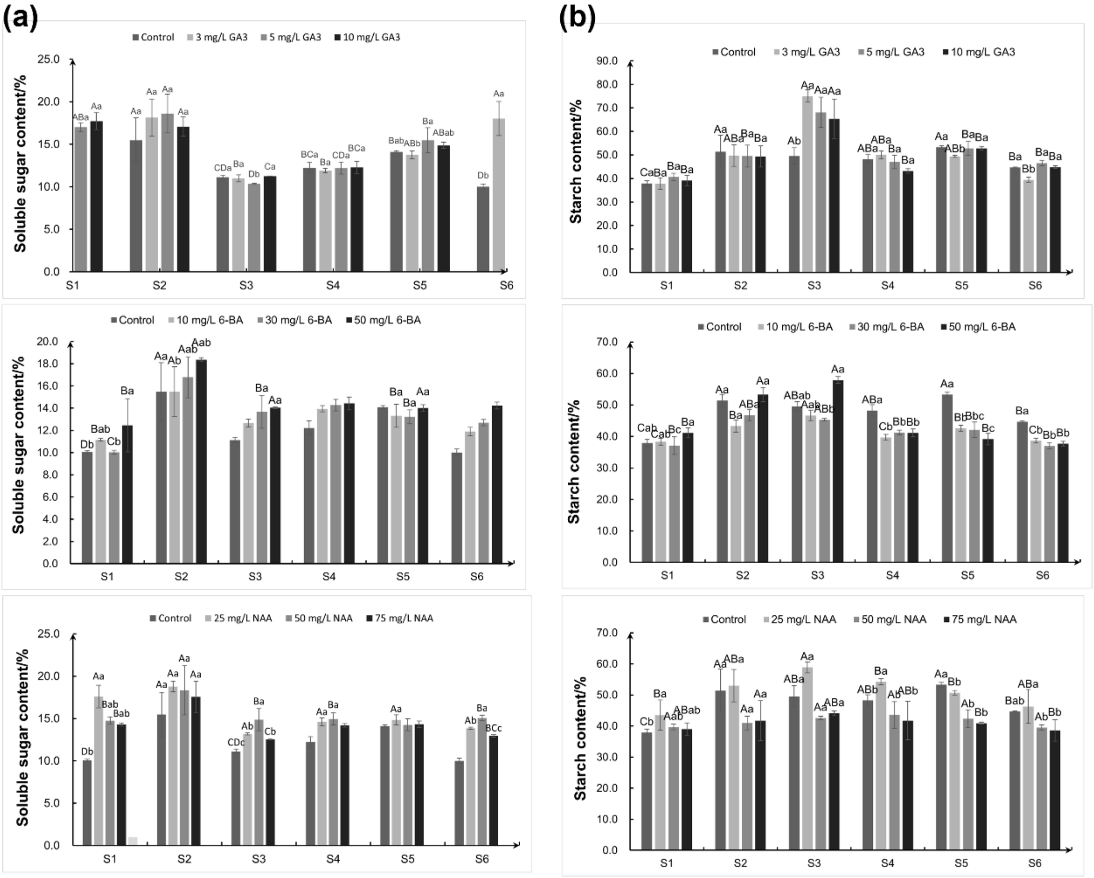

Supplementary Figure S2 Effects of different concentrations of GA_3, 6-BA, and NAA on the accumulation of storage substances in *Ch. utilis* seeds at different developmental stages. Figure a represents the soluble sugar powder content; Figure b represents the starch content. Note: Different capital letters indicate significant differences between the same concentration in different periods, and different lowercase letters indicate significant differences between different concentrations in the same period ($p < 0.05$).
